# From Better Sleep to Improved Mood: A Review of the Biopsychosocial Pathways for Cognitive Behavioral Therapy for Insomnia’s Antidepressant Effects

**DOI:** 10.1007/s40675-026-00376-w

**Published:** 2026-04-30

**Authors:** Ivan Vargas, Harrison Dickens, Arash Assar

**Affiliations:** 1 Department of Psychology, University of Notre Dame, 338 Corbett Family Hall, Notre Dame, Notre Dame, IN 46556 USA; 2 Department of Psychological Science, University of Arkansas, Fayetteville, AR USA

**Keywords:** Insomnia, Depression, Cognitive Behavioral Therapy, Endocrine, Inflammation, Emotion Regulation

## Abstract

**Purpose of Review:**

Recent research supports that Cognitive Behavioral Therapy for Insomnia (CBT-I) improves both insomnia and depression symptoms. The primary purpose of the current review was to summarize potential mechanistic pathways linking insomnia and depression while evaluating how improvements in sleep via CBT-I may lead to reduced depressive symptoms.

**Recent Findings:**

The current literature supports that there are several potential biological, behavioral, and cognitive-affective mechanisms, including improved hypothalamic-pituitary-adrenal (HPA) axis and inflammatory functioning, behavioral activation, and emotion regulation, that may explain the antidepressant effects of CBT-I.

**Summary:**

Critically evaluating the potential mechanisms by which CBT-I improves depression (e.g., HPA-axis, inflammatory, behavioral, and emotional processes) will inform future efforts to enhance the overall effectiveness of CBT-I in patients with comorbid depression. Specifically, it will identify specific mechanisms that can be the focus of more targeted interventions or strategies to streamline CBT-I in patients with depression.

## Introduction

Major depressive disorder is the most common mental health disorder, with a global prevalence of 4.4% (i.e., 322 million people worldwide), and is one of the leading causes of disability [1]. Major depressive disorder is characterized by changes in mood (depressed mood and anhedonia), cognition (e.g. difficulty concentrating, thoughts of worthlessness), and somatic complaints (e.g. fatigue, psychomotor changes) [2]. Chronic insomnia (or Insomnia Disorder) is primarily characterized by difficulties with initiating and/or maintaining sleep lasting over three months and is the most prevalent sleep-wake disorder, with an estimated global prevalence of 12.4% [2, 3]. Untreated insomnia can persist for decades and is a risk factor for multiple medical or psychiatric disorders, particularly depression [4]. Insomnia not only exacerbates depressive symptoms but also increases risk for the onset and recurrence of major depression [5, 6]. Cognitive Behavioral Therapy for Insomnia (CBT-I), the first-line treatment for chronic insomnia [7], reliably improves sleep and shows promising antidepressant effects [8]. However, despite consistent evidence that treating insomnia improves depressive symptoms, the mechanisms through which CBT-I exerts these effects remain poorly understood. The current review summarizes several potential mechanistic pathways linking insomnia and depression and evaluates evidence for how improvements in sleep may reduce depressive symptoms.

## CBT-I as a Treatment for Depression

CBT-I is a highly efficacious, multi-component treatment for insomnia [9–11]. It primarily utilizes behavioral strategies to improve sleep (via strengthening the homeostatic sleep drive and re-establishing a conditioned relationship between a person’s bed and sleep). Meta-analytic estimates indicate large treatment effect sizes, with gains maintained for up to 24 months post-treatment [12, 13]. Studies now also support that CBT-I can reduce symptoms of depression [14–16]. For example, among individuals with comorbid insomnia and depression, CBT-I alone produced a 57% reduction in depression symptom severity (i.e., more than 85% of study participants were no longer depressed at follow-up) [17]. Since then, several studies have further demonstrated that abbreviated or digital CBT-I also is effective in treating depressive symptoms [18, 19]. While these findings are encouraging, systematic evaluations of the overall treatment effects are more mixed. Specifically, recent meta-analytic data suggest that CBT-I has a small-to-moderate effect on depression outcomes [20–22]. Therefore, efforts to identify the mechanisms by which CBT-I improves depressive symptoms may inform when, how, and for whom CBT-I is most beneficial as both a standalone and adjunctive treatment.

## Potential Mechanisms by Which CBT-I Improves Depression Symptoms

**Fig. 1:**
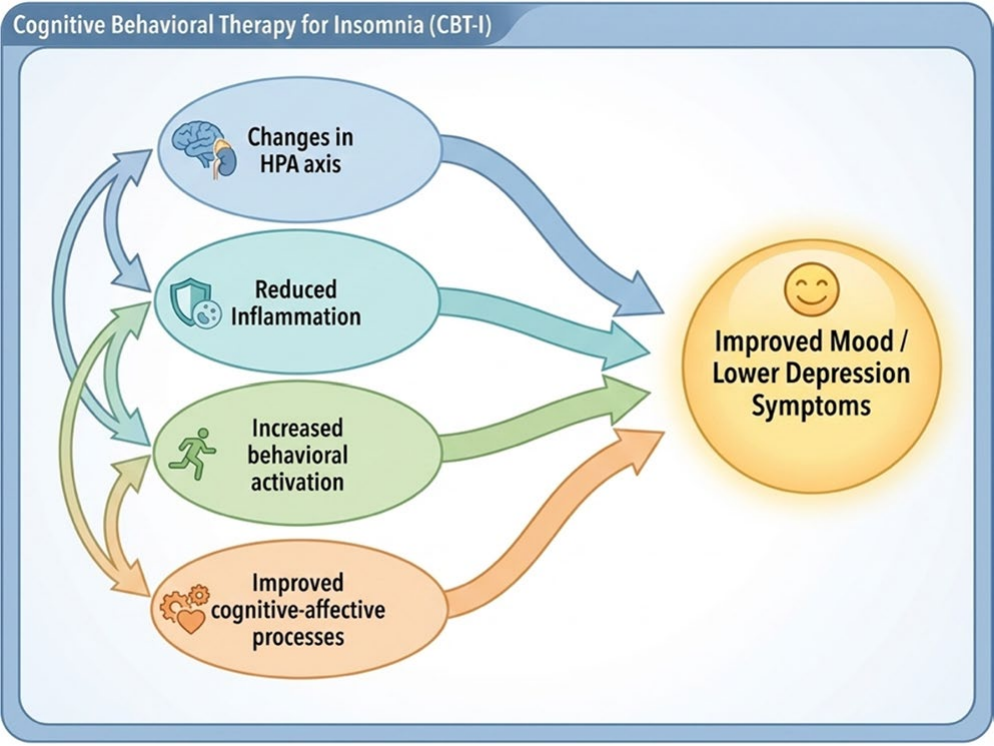
Potential mechanisms by which CBT-I impacts depressive symptoms

Potential Mechanisms by Which CBT-I Improves Depression Symptoms Despite evidence suggesting that CBT-I has at least moderate antidepressant effects, *the mechanisms by which CBT-I reduces depressive symptoms remain relatively unknown* [23, 24]. That said, several pathways are likely, such as improvements in stress and immune system functioning, increased engagement in physical or social activity, and improved cognitive and affective processes (see Fig. 1).

**Changes in hyperarousal**,** stress reactivity**,** and the hypothalamic-pituitary-adrenal (HPA) axis as a potential mechanism.** Improved stress-related regulatory processes may be a potential mechanism by which CBT-I improves depressive symptoms, primarily due to evidence of HPA-axis alterations in both insomnia and depression [25]. Acute stressful life events alone can precipitate the onset of insomnia and depressive symptoms. Importantly, however, insomnia may produce additional stress and impair regulatory processes that in turn may contribute to the development of a depressive episode [26–29]. Specifically, sleep plays an important role in modulating the physiological and psychological processes that respond to, and help us cope with, stress (e.g., down-regulating hormonal and sympathetic responses, up-regulating executive functioning). The HPA axis regulates a series of hormonal responses to acute stressors. These responses culminate in the release of cortisol, which facilitates the mobilization of key adaptive physiological processes such as downregulating digestive activity, maximizing glucose utilization, and promoting sustained alertness/wakefulness [30, 31]. The latter partially explains why exposure to stressors can precipitate *acute* insomnia. That is, deferring sleep during stressful times likely served an important evolutionary function. That said, the association between HPA-axis functioning and sleeplessness is likely bidirectional and may explain why *chronic* insomnia is associated with differences in HPA-axis responses to stress [32–34]. These studies are consistent with the depression literature, which generally supports the notion that depression is associated with an attenuated but prolonged HPA-axis stress response [35, 36].

The HPA-axis is also regulated by circadian processes under basal (non-stressed) conditions. Cortisol levels increase during the night, peak shortly after awakening (i.e., the cortisol awakening response), and decline gradually during the day to reach the lowest levels shortly after sleep onset [37, 38]. In addition to regulating several key bodily functions, cortisol is also responsible for initiating a self-regulatory feedback loop to shut down the HPA-axis [39, 40]. Under normal conditions, this self-regulating process prohibits the system from staying “on” for extended periods, especially during times of stress [41, 42]. That said, the HPA-axis can maintain an elevated state of preparedness to respond to more chronic demands or threats [43–45]. This form of hyperarousal consists of a lower-level tonic activation of the HPA axis [46, 47]. Individuals who work under chronically stressful circumstances (e.g., first responders or military service members), for example, have been shown to exhibit higher levels of tonic cortisol and adrenergic activation [48]. This is also true of individuals with chronic insomnia [49, 50] and major depression [35, 51]. More recently, data from a systematic review and meta-analyses found that chronic insomnia was associated with moderate increases in 24-hour cortisol [52]. While the sleeplessness that comes with insomnia is likely a stressor in itself, increased daytime dysfunction and sleep-related worry may also contribute to maintaining these responses over time. These daily stressors may raise a person’s baseline cortisol levels, particularly at certain times across the 24-hour period (for example, prior to bedtime).

It is also possible that chronic insomnia is related to alterations in hypothalamic regulatory processes (i.e., a central drive problem). For example, at least one study has shown that chronic insomnia is associated with greater levels of corticotropin-releasing hormone (a precursor to cortisol that is produced in the hypothalamus) [53]. This is consistent with other evidence supporting the notion that insomnia is also related to elevated orexin (a neuropeptide produced in the hypothalamus) [54]. Taken together, these data support the possibility that there may be an underlying problem with the modulation of chemical messengers released from the hypothalamus that have important wake-promoting functions (i.e., greater activation at night when wake should be suppressed). If true, this represents one pathway by which insomnia and depression are related and how treatment for insomnia may exert antidepressant effects (via attenuation of tonic activation of the HPA axis and related systems). To date, only one study has examined the impact of CBT-I on HPA-axis functioning [55]. Results from this study indicated that there were no significant differences in HPA-axis functioning between participants receiving four-session CBT-I and controls but did observe differences in the ratio of daytime to nighttime urinary cortisol (less nighttime cortisol) in the CBT-I group. This study was conducted in a medically homogeneous group (i.e., patients with heart failure); therefore, further work is needed to elucidate the interaction between insomnia, depression, and HPA-axis activity.

**Reduced inflammation as a potential mechanism.** The link between sleep and inflammatory processes is also bidirectional. Immune functioning and pro-inflammatory cytokines regulate the sleep-wake drive and acute inflammation can modify the duration and quality of sleep [56]. Alternatively, acute and chronic sleep loss and sleep-related pathology can alter immune functioning. For example, studies have shown that acute sleep loss increases inflammation and patients with chronic insomnia exhibit heightened sympathetic and beta-adrenergic activation [57, 58], which can increase production of pro-inflammatory cytokines [59]. Studies have also shown that insomnia is associated with elevated plasma- and serum-based markers of inflammation [57], especially at night [60]. One interpretation is that insomnia directly elicits inflammatory responses. Over time, increased cytokine production may make the immune system less efficient at handling real or perceived immunological threats [61]. Persistent sleep disturbance may reduce the natural response to antigens, which in turn may lead to greater susceptibility to infection [62] or longer recovery from illness [63]. It is less certain, however, whether improvements in sleep (e.g., via insomnia treatment) are related to changes in inflammatory processes. Some preliminary studies have shown that CBT-I is related to post-treatment decreases in circulating pro-inflammatory cytokines (at least acutely) and reductions in systemic inflammation (lower CRP) [64, 65].

Research has also established that greater cytokine production is observed in individuals with major depression compared to non-depressed individuals [66]. Meta-analytic findings suggest that peripheral cytokine levels are elevated in patients with major depression [67]. Prospective and experimental studies have also found that greater inflammation predicts increases in depressive symptoms and negative mood [68, 69]. In vivo immunological challenges (either via cytokine inducers or vaccines), for example, can lead to acute increases in depressive symptoms, such as low mood, fatigue, and psychomotor slowing [70, 71]. Surprisingly, less is known about the interactive effects of inflammation, sleep disturbance, and depression; however, at least one study in breast cancer survivors has shown that greater sleep disturbance moderated the relationship between inflammation and depressive symptomatology [72]. According to a recently proposed “two-hit” model of depression, insomnia and inflammation jointly contribute to risk for depressive symptoms and this association is likely mediated by affective processes (e.g., reward motivation and emotional recognition) [73]. This is consistent with other theoretical models, such as the Social Signal Transduction Theory of Depression, and research supporting that executive control processes mediate the link between inflammatory responses and depressive symptoms [74]. These theoretical models can inform empirical efforts to understand how increases in inflammation are directly related to having insomnia. They also provide a framework for understanding whether reductions in inflammation that occur via treatment for insomnia may help explain the antidepressant effects that are often observed in patients with comorbid insomnia and depression.

**Increased behavioral activation as a potential mechanism.** Increased behavioral activation may represent one potential mechanism linking improvements in sleep to reductions in depressive symptoms. Behavioral activation, an empirically supported psychosocial treatment for MDD, aims to reduce depression by increasing engagement in meaningful, rewarding, and goal-directed activities (often those associated with pleasure or mastery) while decreasing engagement in activities that maintain depression or exacerbate depression [75]. Meta-analytic evidence further supports behavioral activation as an effective intervention for reducing depressive symptoms [76]. In this context, improvements in sleep may lead to increased daytime energy and motivation, thereby facilitating greater engagement in meaningful, goal-directed activities, which is a core principle of behavioral activation. Two common features of these activities are that they promote physical activity and social connectedness. A robust body of evidence shows that higher levels of physical activity (PA) and social connectedness are associated with lower depressive symptoms, whereas physical inactivity, social withdrawal, and loneliness predict poorer mood outcomes [77, 78]. Because insomnia is characterized by fatigue, low energy, and reduced cognitive and emotional bandwidth, successful CBT-I may remove key barriers to activity engagement. Physically active individuals are less likely to report symptoms of depression [79, 80]. Similarly, individuals who report a lack of social connectedness or support are more likely to endorse depressive symptoms [78], and low social connectedness has been identified as a significant risk factor for suicidality [81]. Relatedly, lower PA and greater sleep loss have also been linked to greater loneliness and social isolation [82, 83], suggesting that both good sleep and regular PA may be protective against the negative effects of social isolation. These findings, in general, emphasize the importance of being physically active and socially connected and the role this plays in building resilience to depression, a link also observed during the COVID-19 pandemic, during which reduced PA was associated with greater depressive symptoms [84].

The mechanism here may be related to the notion that engaging in PA is a form of behavioral activation, and therefore, the overall effects of negative mood can be attenuated by exercising or being physically active. Greater PA may also impact physiological and hormonal processes that have been implicated in the regulation of mood. For example, aerobic exercise increases serotonin and dopamine concentrations in the brain [85], leading to more positive emotional processing and reduced depressive symptoms [86]. PA can also regulate the release of corticotropin-releasing hormone from the hypothalamus and adrenocorticotropic hormone from the pituitary gland, subsequently decreasing cortisol levels as glucocorticoid receptor sensitivity increases post-exercise [87]. Taken together, these findings converge on a single important point: one plausible mechanism by which CBT-I improves depression symptom severity is via increases in PA and improvements in daytime social functioning or opportunities for social engagement [88, 89]. Individuals who are sleeping better may have more energy and greater motivation to engage in these activities.

**Improvements in cognitive processes as potential mechanism.** Insomnia is often characterized by maladaptive thinking patterns (e.g., dysfunctional sleep-related beliefs), heightened cognitive arousal, and repetitive negative thinking [90]. Although CBT-I directly targets sleep-related behaviors and cognitions, broader improvements in worry, rumination, and other cognitive and psychological processes may also occur [91]. These secondary cognitive and emotional shifts may help explain the reductions in depressive symptoms observed following CBT-I.

Repetitive negative thinking features prominently in seminal models of insomnia and contributes to daytime impairment, including depression and anxiety [92]. It is not surprising, then, that several studies have examined whether reductions in repetitive negative thinking following CBT-I are associated with lower depression or anxiety. Findings from a systematic review and meta-analysis support moderate-to-large reductions in worry following CBT-I but only small decreases in rumination [93]. Importantly, this review did not identify clear evidence that reductions in repetitive negative thinking explained post-treatment improvements in depression and anxiety symptoms. The authors concluded that CBT-I may exert stronger effects on *sleep-specific* forms of repetitive negative thinking (e.g., pre-sleep worry) than on *rumination*. This distinction has implications for CBT-I’s antidepressant effects: if CBT-I primarily reduces insomnia-related negative thinking rather than general rumination, its impact on depressive symptoms may be more modest. That said, another study found that even reductions in dysfunctional beliefs about sleep can mediate the effect of CBT-I on depressive symptoms [94]. More recent findings provide a somewhat more nuanced picture. Specifically, reductions in depressive symptoms following CBT-I have been linked to improvements in rumination [95–97].

A separate line of research also implicates improvements in executive functioning as a potential pathway through which CBT-I may alleviate depressive symptoms. Although executive control has not yet been formally tested as a mechanism of CBT-I’s antidepressant effects, previous trials provide indirect evidence. One RCT demonstrated that digital CBT-I produced robust and sustained improvements in self-reported cognitive impairment, though not on objective cognitive performance tests, with effects maintained for up to 6 months [98]. Similarly, an RCT among individuals with fibromyalgia found that CBT-I led to greater improvements in alertness and executive functioning [99]. While the literature is limited, early improvements in executive function during antidepressant medication treatment have been associated with more favorable treatment responses [100]. Collectively, these findings raise the possibility that CBT-I may indirectly support reductions in depression by strengthening cognitive control systems that regulate attention away from negative internal states (e.g., rumination, worry).

Taken together, improvements in cognitive and psychological processes, including reductions in repetitive negative thinking, changes in dysfunctional beliefs, and improvements in executive control, represent promising mechanistic pathways through which CBT-I may influence depressive outcomes. However, evidence remains mixed, particularly regarding rumination, and the extent to which CBT-I impacts transdiagnostic versus sleep-specific cognitive processes remains an open question. Future work is needed to clarify these distinctions and to determine whether incorporating strategies targeting repetitive negative thinking (including rumination), dysfunctional sleep-related cognitions, or executive control may augment the antidepressant effects of CBT-I.

**Improvements in affective functioning as a potential mechanism.** There is a bidirectional link between sleep and affective functioning, specifically emotion, emotion regulation, and mood. Emotion regulation is a series of processes intended to shape emotional experience including what emotions are experienced, when emotions occur, and how emotions are expressed [101]. Watling and colleagues (2017) proposed a theoretical model that highlighted the bidirectional relationship between sleep and mood, and the proposed role of emotion and emotion regulation in explaining this association [102]. Based on this model, emotion and emotion regulation are considered the building blocks for mood (and therefore mood disorders like depression). Improvements in emotion regulation, therefore, could be a mechanism by which CBT-I improves depressive outcomes.

Most of the evidence on the influence of impaired sleep on emotion is derived from studies using experimental sleep paradigms (e.g. total sleep deprivation) and reactions to emotionally-laden stimuli (e.g. emotion-invoking images). Data from these studies show that sleep loss is related to increased emotional intensity and volatility [103], as well as more negatively valenced emotional reactions to neutral and negative stimuli [104, 105]. These findings were also accompanied by simultaneous alterations in amygdala activation and decreases in medial prefrontal cortex activation for those in sleep deprivation conditions [103, 104]. Despite a clear link between impaired sleep, emotion, and depression, findings on improvements in emotion or emotional reactivity during the course of CBT-I are mixed [106–108]. For example, an RCT of 205 individuals diagnosed with depression and chronic insomnia undergoing digital CBT-I treatment did not find a significant improvement in emotional stimulus recognition (both positive and negative), despite seeing meaningful reductions in both insomnia and depression [109].

Emotion regulation strategies have also been implicated in the onset and maintenance of both chronic insomnia and depression. While determining whether an emotion regulation strategy is adaptive varies based on several individual and contextual factors, research evidence suggests that strategies such as disengagement (e.g. avoiding or suppressing emotion) or cognitive perseveration (e.g. repetitive negative thinking) are likely to be maladaptive, especially if used habitually [110]. Those with insomnia have been shown to exhibit higher rates of disengagement strategies (cognitive or experiential avoidance) and lower rates of adaptive engagement (e.g. cognitive reappraisal) [111–113]. Experimental studies primarily conducted in youth also support that sleep-restricted individuals tend to rely less on adaptive emotion regulation strategies; however, there were no differences in the likelihood that they would use maladaptive emotion regulation strategies [114]. Furthermore, no studies have investigated whether the alterations in emotion regulation that are observed with sleep disturbance directly contribute to future depression or whether behavioral sleep interventions lead to greater engagement with adaptive emotional regulation strategies.

Several studies, however, have examined the added benefit of incorporating an emotion regulation module (e.g. relaxation skills, emotion-related psychoeducation, acceptance) into CBT-I, providing some insight into the role of emotion regulation in alleviating both insomnia and depression. Evidence on the added effectiveness of emotion regulation modules has been mixed. One study focused on a small sample of those with insomnia found that CBT-I plus emotion regulation training was no more effective in reducing insomnia symptoms or improving use of emotion regulation strategies compared to traditional CBT-I [115]. In contrast, a digital version of CBT-I plus emotion regulation training delivered to a sample of participants with insomnia and mild anxiety or depression showed significant improvements in insomnia, depression, and anxiety symptoms compared to a waitlist control group [116]. Despite the effectiveness of this intervention, the added emotion regulation components were not compared directly to the original version of CBT-I, limiting insight into the unique role of emotion regulation in improving both insomnia and depression. Future studies incorporating emotion regulation modules into CBT-I would benefit from the inclusion of a CBT-I–only group to establish the unique benefit of emotion regulation on both insomnia and depression.

## Strengths, Limitations, & Future Directions

The literature reviewed has some notable strengths. First, the candidate mechanisms span biological, behavioral, and cognitive-affective domains, providing a multi-level framework for understanding the mechanisms by which CBT-I exerts its antidepressant effects. Second, the evidence base also draws on a number of methodological approaches, including randomized controlled trials, experimental sleep paradigms, and meta-analytic studies, which provides well-rounded and strengthened overall plausibility of the proposed mechanisms. Third, the inclusion of both traditional and digital CBT-I formats, as well as diverse populations, broadens the clinical applicability of these findings.

That said, there are several important limitations. Perhaps most critically, very few studies have used prospective studies and formal mediation analyses to assess whether the proposed mechanisms explain the post-treatment reductions in depressive symptoms following CBT-I. Much of the evidence remains indirect and relies on parallel but separate literature on insomnia, depression, and the proposed mediating processes. For example, while research has established that both insomnia and depression are independently associated with HPA-axis dysregulation and elevated inflammation, few studies have examined whether treating insomnia with CBT-I actually changes these processes, and fewer have examined whether such changes explain improvement in depressive symptoms. Similarly, while preliminary studies suggest that CBT-I may reduce circulating pro-inflammatory cytokines and systemic inflammation [64, 65], no studies have tested whether these reductions are what drive the improvements in depressive symptoms that are often observed after treatment.

Furthermore, much of what we know about the influence of impaired sleep on emotion and emotion regulation comes from experimental sleep deprivation studies conducted in healthy samples, which may not generalize to individuals with chronic insomnia or comorbid insomnia and depression. For example, findings that sleep loss increases emotional intensity and negativity bias toward neutral and negative stimuli were observed under total sleep deprivation conditions, which differ meaningfully from the partial and fragmented sleep patterns that characterize chronic insomnia [117–119]. Similarly, many experimental studies on emotion regulation under sleep restricted conditions have been conducted exclusively in youth samples [120], further limiting generalizability.

Findings on several of the proposed cognitive-affective mechanisms have also been mixed. With respect of rumination, meta-analytic evidence supports only small reductions following CBT-I, and there is no clear evidence that these reductions explain improvements in depression [121], though more recent individual studies have linked reductions in rumination post–CBT-I to improvements in depressive symptoms [96, 122]. Findings on several other proposed mechanisms such as emotional reactivity, and the added benefits of emotion regulation modules to CBT-I, have also been mixed. Additionally, variability in CBT-I delivery format, treatment dose, and outcome measurement across studies make it difficult to draw definitive conclusions about mechanism-specific effects.

With respect to these limitations, several lines of future research are needed to clarify the mechanistic pathways by which CBT-I improves depressive symptoms. Most importantly future research incorporating formal mediation analyses are needed. For example, future studies should test whether changes in HPA-axis functioning, inflammatory processes, behavioral activation, repetitive negative thinking, and emotion regulation statistically account for reductions in depressive symptoms following CBT-I. Furthermore, longitudinal designs with repeated assessments of both proposed mediators and depression outcomes across the course of treatment would help establish temporal ordering and strengthen causal inferences. For example, studies could examine whether early reductions in pre-bedtime cortisol or proinflammatory cytokines precede later improvements in depressive symptoms, or whether early increases in daytime physical activity and social engagement occur before changes in mood. Similarly, assessing whether reductions in rumination or pre-sleep cognitive arousal during the early sessions of CBT-I predict subsequent decreases in depressive symptom severity would help clarify whether these cognitive-affective processes function as mechanisms of change or simply co-occur with improvements in sleep. That is, without assessing these processes at separate time points, it remains unclear whether the proposed mediators actually drive the antidepressant effects of CBT-I, whether they change as a result of improvements in depression, or whether both improve simultaneously due to shared underlying factors. Finally, future research should seek to identify the role of variables that moderate this link, such as baseline insomnia or depression symptom severity, gender, or age, to see for whom CBT-I is most effective in reducing depressive symptoms.

## Conclusion

CBT-I likely exerts its antidepressant effects through a complex interplay of physiological, behavioral, and cognitive-affective pathways. These include, but are likely not limited to, modulating HPA-axis functioning, reducing systemic inflammation, facilitating behavioral activation, and improving emotion regulation. While current evidence supports that improving insomnia symptoms can improve depressive symptoms, further research is required to isolate which specific mechanisms are most influential. Ultimately, elucidating these pathways will not only refine our understanding of the insomnia-depression link, but also enhance the precision and efficacy of transdiagnostic treatments for comorbid insomnia and depression.

##  Key References

- Tamm S, Tse KYK, Hellier J, Saunders KEA, Harmer CJ, Espie CA, et al. Emotional Processing Following Digital Cognitive Behavioral Therapy for Insomnia in People With Depressive Symptoms: A Randomized Clinical Trial. JAMA Netw Open. 2025 Feb 27;8(2):e2461502.
  - ◦In adults with insomnia disorder and clinically significant depressive symptoms, those assigned to 6-session digital CBT-I, compared to a sleep hygiene control condition, experienced significant improvements in both insomnia and depression symptoms. These effects were in part due to reductions in negative affect, emotion regulation difficulties, and worry, but not changes in negative bias in recognizing happy or sad facial expressions.
- Yu K, Xia L, Chen HH, Zou TT, Zhang Y, Zhang P, et al. Association Between Sleep Reactivity, Pre-Sleep Arousal State, and Neuroendocrine Hormones in Patients with Chronic Insomnia Disorder. Nature and Science of Sleep. 2024 Dec 31;16:1907–19.
  - ◦Compared to health controls, patients with chronic insomnia disorder reported greater sleep reactivity and pre-sleep arousal, which were significantly associated with elevated serum cortisol, corticotropin-releasing hormone, and copeptin. These data support that elevated neuroendocrine activity may be a key pathway linking cognitive–physiological hyperarousal to insomnia.
- Li SH, Corkish B, Richardson C, Christensen H, Werner-Seidler A. The role of rumination in the relationship between symptoms of insomnia and depression in adolescents. Journal of Sleep Research. 2024;33(2):e13932.
  - ◦A series of mediation analyses revealed that rumination, but not unhelpful beliefs about sleep, partly explained the link between insomnia and depression symptoms at baseline. This study also provided some initial support that reductions in rumination were associated with decreases in depressive symptoms following use of a mobile CBT-I intervention.
- Lau PH, Carney AE, Marway OS, Carmona NE, Amestoy M, Carney CE. Investigating the antidepressant effects of CBT-I in those with major depressive and insomnia disorders. Journal of Affective Disorders Reports. 2022 July 1;9:100366.
  - ◦In adults with major depression and chronic insomnia, brief four-session CBT-I produced significant improvements in depressive symptoms, with greater treatment gains observed in younger participants, those with less severe baseline depression, and those who a lower their tendency to ruminate (particularly about daytime fatigue). A limitation of the study was the lack of a control group.

## Data Availability

No datasets were generated or analysed during the current study.

## References

[1] World Health Organization. Depression and Other Common Mental Disorders: Global Health Estimates [Internet]. 2017. https://apps.who.int/iris/bitstream/handle/10665/254610/WHO-MSD-MER2017.2-eng.pdf

[2] American Psychiatric Association. (2022). *Diagnostic and statistical manual of mental disorders* (5th ed., text rev.). 10.1176/appi.books.9780890425787

[3] van Straten A, Weinreich KJ, Fábián B, Reesen J, Grigori S, Luik AI, et al. The prevalence of insomnia disorder in the general population: a meta-analysis. J Sleep Res. 2025;n/a:e70089. 10.1111/jsr.70089. 10.1111/jsr.70089 PMC12426706 40369835

[4] Hertenstein E, Benz F, Schneider C, Baglioni C. Insomnia—a risk factor for mental disorders. J Sleep Res. 2023;e13930. 10.1111/JSR.13930. 37211915 10.1111/jsr.13930

[5] Baglioni C, Battagliese G, Feige B, Spiegelhalder K, Nissen C, Voderholzer U, et al. Insomnia as a predictor of depression: a meta-analytic evaluation of longitudinal epidemiological studies. J Affect Disord. 2011;135:10–9. 10.1016/j.jad.2011.01.011. 21300408 10.1016/j.jad.2011.01.011

[6] Li L, Wu C, Gan Y, Qu X, Lu Z. Insomnia and the risk of depression: a meta-analysis of prospective cohort studies. BMC Psychiatry. 2016. 10.1186/s12888-016-1075-3. 27816065 10.1186/s12888-016-1075-3 PMC5097837

[7] Qaseem A, Kansagara D, Forciea MA, Cooke M, Denberg TD, Barry MJ. Management of chronic insomnia disorder in adults: a clinical practice guideline from the American College of Physicians. Ann Intern Med. 2016;165:125–33. 10.7326/M15-2175. 27136449 10.7326/M15-2175

[8] Cunningham JEA, Shapiro CM. Cognitive behavioural therapy for insomnia (CBT-I) to treat depression: a systematic review. J Psychosom Res. 2018;106:1–12. 10.1016/J.JPSYCHORES.2017.12.012. 29455893 10.1016/j.jpsychores.2017.12.012

[9] Irwin MR, Cole JC, Nicassio PM. Comparative meta-analysis of behavioral interventions for insomnia and their efficacy in middle-aged adults and in older adults 55 + years of age. Health Psychol. 2006;25:3–14. 10.1037/0278-6133.25.1.3. 16448292 10.1037/0278-6133.25.1.3

[10] Mitchell MD, Gehrman P, Perlis M, Umscheid CA. Comparative effectiveness of cognitive behavioral therapy for insomnia: a systematic review. BMC Fam Pract. 2012. 10.1186/1471-2296-13-40. 22631616 10.1186/1471-2296-13-40 PMC3481424

[11] Smith MT, Perlis ML, Park A, Smith MS, Pennington JM, Giles DE, et al. Comparative meta-analysis of pharmacotherapy and behavior therapy for persistent insomnia. Am J Psychiatry. 2002;159:5–11. 10.1176/appi.ajp.159.1.5. 11772681 10.1176/appi.ajp.159.1.5

[12] van der Zweerde T, Bisdounis L, Kyle SD, Lancee J, van Straten A. Cognitive behavioral therapy for insomnia: a meta-analysis of long-term effects in controlled studies. Sleep Med Rev. 2019. 10.1016/j.smrv.2019.08.002. 31491656 10.1016/j.smrv.2019.08.002

[13] Morin CM, Vallieres A, Guay B, Ivers H, Savard J, Merette C, et al. Cognitive behavioral therapy, singly and combined with medication, for persistent insomnia: a randomized controlled trial. JAMA. 2009;301:2005–15. 10.1001/jama.2009.682. 19454639 10.1001/jama.2009.682 PMC3050624

[14] Carney CE, Edinger JD, Kuchibhatla M, Lachowski AM, Bogouslavsky O, Krystal AD, et al. Cognitive behavioral insomnia therapy for those with insomnia and depression: a randomized controlled clinical trial. Sleep. 2017;40:1–13. 10.1093/sleep/zsx019. 10.1093/sleep/zsx019 PMC5806549 28199710

[15] Manber R, Buysse DJ, Edinger J, Krystal A, Luther JF, Wisniewski SR, et al. Efficacy of cognitive-behavioral therapy for insomnia combined with antidepressant pharmacotherapy in patients with comorbid depression and insomnia: a randomized controlled trial. J Clin Psychiatry. 2016. 10.4088/JCP.15m10244. 27788313 10.4088/JCP.15m10244

[16] Ashworth DK, Sletten TL, Junge M, Simpson K, Clarke D, Cunnington D, et al. A randomized controlled trial of cognitive behavioral therapy for insomnia: an effective treatment for comorbid insomnia and depression. J Couns Psychol. 2015;62:115–23. 10.1037/cou0000059. 25867693 10.1037/cou0000059

[17] Taylor DJ, Lichstein KL, Weinstock J, Sanford S, Temple JR. A pilot study of cognitive-behavioral therapy of insomnia in people with mild depression. Behav Ther. 2007;38:49–57. 10.1016/j.beth.2006.04.002. 17292694 10.1016/j.beth.2006.04.002

[18] Cheng P, Luik AI, Fellman-Couture C, Peterson E, Joseph CLM, Tallent G, et al. Efficacy of digital CBT for insomnia to reduce depression across demographic groups: a randomized trial. Psychol Med. 2019;49:491–500. 10.1017/S0033291718001113. 29792241 10.1017/S0033291718001113 PMC7050476

[19] Wagley JN, Rybarczyk B, Nay WT, Danish S, Lund HG. Effectiveness of abbreviated CBT for insomnia in psychiatric outpatients: sleep and depression outcomes. J Clin Psychol. 2013;69:1043–55. 10.1002/jclp.21927. 23109266 10.1002/jclp.21927

[20] Gebara MA, Siripong N, DiNapoli EA, Maree RD, Germain A, Reynolds CF, et al. Effect of insomnia treatments on depression: a systematic review and meta-analysis. Depress Anxiety. 2018;35:717–31. 10.1002/DA.22776. 29782076 10.1002/da.22776

[21] Lee S, Oh JW, Park KM, Lee S, Lee E. Digital cognitive behavioral therapy for insomnia on depression and anxiety: a systematic review and meta-analysis. npj Digit Med. 2023;6:52. 10.1038/s41746-023-00800-3. 36966184 10.1038/s41746-023-00800-3 PMC10039857

[22] Ye YY, Chen NK, Chen J, Liu J, Lin L, Liu YZ, et al. Internet-based cognitive-behavioural therapy for insomnia (ICBT-i): a meta-analysis of randomised controlled trials. BMJ Open. 2016;6:1–10. 10.1136/bmjopen-2015-010707. 10.1136/bmjopen-2015-010707 PMC5168528 27903557

[23] Riemann D, Voderholzer U. Primary insomnia: a risk factor to develop depression? J Affect Disord. 2003;76:255–9. 10.1016/S0165-0327(02)00072-1. 12943956 10.1016/s0165-0327(02)00072-1

[24] Vargas I, Perlis ML. Insomnia and depression: clinical associations and possible mechanistic links. Curr Opin Psychol. 2020;34:95–9. 10.1016/j.copsyc.2019.11.004. 31846870 10.1016/j.copsyc.2019.11.004 PMC12172691

[25] Asarnow LD. Depression and sleep: what has the treatment research revealed and could the HPA axis be a potential mechanism? Curr Opin Psychol. 2020;34:112–6. 10.1016/J.COPSYC.2019.12.002. 31962280 10.1016/j.copsyc.2019.12.002 PMC8412030

[26] Gregory AM, Caspi A, Moffitt TE, Poulton R. Family conflict in childhood: a predictor of later insomnia. Sleep. 2006;29:1063–7. 16944675 10.1093/sleep/29.8.1063

[27] Hall M, Buysse DJ, Nofzinger E, Reynolds CF, Thompson W, Mazumdar S, et al. Financial strain is a significant correlate of sleep continuity disturbances in late-life. Biol Psychol. 2008;77:217–22. 10.1016/j.biopsycho.2007.10.012. 18055094 10.1016/j.biopsycho.2007.10.012 PMC2267650

[28] Mezick EJ, Matthews K, Hall M, Kamarck TW, Buysse DJ, Owens JF, et al. Intra-individual variability in sleep duration and fragmentation: associations with stress. Psychoneuroendocrinology. 2009;34:1346–54. 10.1016/j.psyneuen.2009.04.005. 19450933 10.1016/j.psyneuen.2009.04.005 PMC2743778

[29] Pillai V, Roth T, Mullins HM, Drake CL. Moderators and mediators of the relationship between stress and insomnia: stressor chronicity, cognitive intrusion, and coping. Sleep. 2014;37:1199–208. 10.5665/sleep.3838. 25061248 10.5665/sleep.3838 PMC4098805

[30] de Kloet ER. Brain corticosteroid receptor balance and homeostatic control. Front Neuroendocrinol. 1991;12:95–164. 10.1080/09614520701469617.

[31] Johnson EO, Kamilaris TC, Chrousos GP, Gold PW. Mechanisms of stress: a dynamic overview of hormonal and behavioral homeostasis. Neurosci Biobehav Rev. 1992;16:115–30. 1630726 10.1016/s0149-7634(05)80175-7

[32] Chen IY, Jarrin DC, Ivers H, Morin CM. Investigating psychological and physiological responses to the Trier Social Stress Test in young adults with insomnia. Sleep Med. 2017;40:11–22. 10.1016/j.sleep.2017.09.011. 29221772 10.1016/j.sleep.2017.09.011

[33] Vgontzas AN, Fernandez-Mendoza J, Lenker KP, Basta M, Bixler EO, Chrousos GP. Hypothalamic–pituitary–adrenal (HPA) axis response to exogenous corticotropin-releasing hormone (CRH) is attenuated in men with chronic insomnia. J Sleep Res. 2022;31:e13526. 10.1111/JSR.13526. 34825417 10.1111/jsr.13526

[34] Devine JK, Bertisch SM, Yang H, Scott-Sutherland J, Wilkins A, Molina V, et al. Glucocorticoid and inflammatory reactivity to a repeated physiological stressor in insomnia disorder. Neurobiol Sleep Circadian Rhythms. 2019;6:77–84. 10.1016/J.NBSCR.2018.06.001. 31236523 10.1016/j.nbscr.2018.06.001 PMC6586925

[35] Burke HM, Davis MC, Otte C, Mohr DC. Depression and cortisol responses to psychological stress: a meta-analysis. Psychoneuroendocrinology. 2005;30:846–56. 15961250 10.1016/j.psyneuen.2005.02.010

[36] Lopez-Duran NL, McGinnis E, Kuhlman K, Geiss E, Vargas I, Mayer S. HPA-axis stress reactivity in youth depression: evidence of impaired regulatory processes in depressed boys. Stress. 2015;18:545–53. 10.3109/10253890.2015.1053455. 26115161 10.3109/10253890.2015.1053455 PMC5403248

[37] Buckley TM, Schatzberg AF. On the interactions of the hypothalamic-pituitary-adrenal (HPA) axis and sleep: normal HPA axis activity and circadian rhythm, exemplary sleep disorders. J Clin Endocrinol Metab. 2005;90:3106–14. 10.1210/jc.2004-1056. 15728214 10.1210/jc.2004-1056

[38] Gunnar MR, Vazquez DM. Low cortisol and a flattening of expected daytime rhythm: potential indices of risk in human development. Dev Psychopathol. 2001;13:515–38. 11523846 10.1017/s0954579401003066

[39] Gunnar M, Quevedo K. The neurobiology of stress and development. Annu Rev Psychol. 2007;58:145–73. 10.1146/annurev.psych.58.110405.085605. 16903808 10.1146/annurev.psych.58.110405.085605

[40] Tsigos C, Chrousos GP. Physiology of the hypothalamic-pituitary-adrenal axis in health and dysregulation in psychiatric and autoimmune disorders. Endocrinol Metab Clin North Am. 1994;23:451–66. 7805648

[41] Jones MT, Tiptaft EM, Brush FR, Fergusson DA, Neame RL. Evidence for dual corticosteroid receptor mechanisms in the feedback control of adrenocorticotrophin secretion. J Endocrinol. 1974;60:223–33. 10.1677/joe.0.0600223. 4361174 10.1677/joe.0.0600223

[42] Myers B, McKlveen JM, Herman JP. Neural regulation of the stress response: the many faces of feedback. Cell Mol Neurobiol. 2012. 10.1007/s10571-012-9801-y. 22302180 10.1007/s10571-012-9801-y PMC3956711

[43] Jacobs N, Myin-Germeys I, Derom C, Delespaul P, van Os J, Nicolson NA. A momentary assessment study of the relationship between affective and adrenocortical stress responses in daily life. Biol Psychol. 2007;74:60–6. 10.1016/j.biopsycho.2006.07.002. 16942831 10.1016/j.biopsycho.2006.07.002

[44] McHale SM, Blocklin MK, Walter KN, Davis KD, Almeida DM, Klein LC. The role of daily activities in youths’ stress physiology. J Adolesc Health. 2012;51:623–8. 10.1016/j.jadohealth.2012.03.016. 23174474 10.1016/j.jadohealth.2012.03.016 PMC3532943

[45] Smyth J, Ockenfels MC, Porter L, Kirschbaum C, Hellhammer DH, Stone AA. Stressors and mood measured on a momentary basis are associated with salivary cortisol secretion. Psychoneuroendocrinology. 1998;23:353–70. 10.1016/S0306-4530(98)00008-0. 9695136 10.1016/s0306-4530(98)00008-0

[46] Wassing R, Benjamins JS, Dekker K, Moens S, Spiegelhalder K, Feige B, et al. Slow dissolving of emotional distress contributes to hyperarousal. Proc Natl Acad Sci U S A. 2016;113:2538–43. 10.1073/pnas.1522520113. 26858434 10.1073/pnas.1522520113 PMC4780629

[47] Wassing R, Benjamins JS, Talamini LM, Schalkwijk F, Someren EJWV. Overnight worsening of emotional distress indicates maladaptive sleep in insomnia. Sleep. 2019;42:1–8. 10.1093/sleep/zsy268. 10.1093/sleep/zsy268 30590834

[48] Walker A, McKune A, Ferguson S, Pyne DB, Rattray B. Chronic occupational exposures can influence the rate of PTSD and depressive disorders in first responders and military personnel. Extreme Physiol Med. 2016;5. 10.1186/S13728-016-0049-X. 10.1186/s13728-016-0049-x PMC4947320 27429749

[49] Floam S, Simpson N, Nemeth E, Scott-Sutherland J, Gautam S, Haack M. Sleep characteristics as predictor variables of stress systems markers in insomnia disorder. J Sleep Res. 2015;24:296–304. 10.1111/jsr.12259. 25524529 10.1111/jsr.12259

[50] Vgontzas AN, Bixler EO, Lin HM, Prolo P, Mastorakos G, Vela-Bueno A, et al. Chronic insomnia is associated with nyctohemeral activation of the Hypothalamic-Pituitary-Adrenal Axis: clinical implications. J Clin Endocrinol Metab. 2001;86:3787–94. 11502812 10.1210/jcem.86.8.7778

[51] Morris MC, Rao U. Cortisol response to psychosocial stress during a depressive episode and remission. Stress. 2014;17:51–8. 10.3109/10253890.2013.857398. 24144001 10.3109/10253890.2013.857398 PMC3920542

[52] Dressle RJ, Feige B, Spiegelhalder K, Schmucker C, Benz F, Mey NC, et al. HPA axis activity in patients with chronic insomnia: a systematic review and meta-analysis of case–control studies. Sleep Med Rev. 2022;62:101588. 10.1016/J.SMRV.2022.101588. 35091194 10.1016/j.smrv.2022.101588

[53] Yu K, Xia L, Chen H-H, Zou T-T, Zhang Y, Zhang P, et al. Association between sleep reactivity, pre-sleep arousal state, and neuroendocrine hormones in patients with chronic insomnia disorder. Nat Sci Sleep. 2024;16:1907–19. 10.2147/NSS.S491040. 39655316 10.2147/NSS.S491040 PMC11627101

[54] Krystal AD, Benca MRM, Kilduff TS, et al. Understanding the sleep-wake cycle: sleep, insomnia, and the orexin system. J Clin Psychiatry. 2013;74:21876. 10.4088/JCP.13011SU1C. 10.4088/JCP.13011su1c 24107804

[55] Redeker NS, Conley S, Anderson G, Cline J, Andrews L, Mohsenin V, et al. Effects of cognitive behavioral therapy for insomnia on sleep, symptoms, stress, and autonomic function among patients with heart failure. Behav Sleep Med. 2020;18:190–202. 10.1080/15402002.2018.1546709. 30461315 10.1080/15402002.2018.1546709 PMC6529289

[56] Mullington J, Korth C, Hermann DM, Orth A, Galanos C, Holsboer F, et al. Dose-dependent effects of endotoxin on human sleep. Am J Physiol Regul Integr Comp Physiol. 2000;278:R947-55. 10.1152/ajpregu.2000.278.4.R947. 10749783 10.1152/ajpregu.2000.278.4.R947

[57] Irwin MR, Olmstead R, Carroll JE. Sleep disturbance, sleep duration, and inflammation: a systematic review and meta-analysis of cohort studies and experimental sleep deprivation. Biol Psychiatry. 2016;80:40–52. 10.1016/j.biopsych.2015.05.014. 26140821 10.1016/j.biopsych.2015.05.014 PMC4666828

[58] Bonnet MH, Arand DL. Hyperarousal and insomnia: state of the science. Sleep Med Rev. 2010;14:97–108. 10.1016/j.smrv.2009.05.002. 19640748 10.1016/j.smrv.2009.05.002

[59] Goebel MU, Mills PJ, Irwin MR, Ziegler MG. Interleukin-6 and Tumor Necrosis Factor-α production after acute psychological stress, exercise, and infused Isoproterenol: differential effects and pathways. Psychosom Med. 2000;62:591–8. 10.1097/00006842-200007000-00019. 10949106 10.1097/00006842-200007000-00019

[60] Burgos I, Richter L, Klein T, Fiebich B, Feige B, Lieb K, et al. Increased nocturnal interleukin-6 excretion in patients with primary insomnia: a pilot study. Brain Behav Immun. 2006;20:246–53. 10.1016/j.bbi.2005.06.007. 16084689 10.1016/j.bbi.2005.06.007

[61] Taylor DJ, Kelly K, Kohut ML, Song KS. Is insomnia a risk factor for decreased Influenza vaccine response? Behav Sleep Med. 2017;15:270–87. 10.1080/15402002.2015.1126596. 27077395 10.1080/15402002.2015.1126596 PMC5554442

[62] Prather AA, Janicki-Deverts D, Hall MH, Cohen S. Behaviorally assessed sleep and susceptibility to the common cold. Sleep. 2015;38:1353–9. 10.5665/sleep.4968. 26118561 10.5665/sleep.4968 PMC4531403

[63] Vargas I, Muench A, Grandner MA, Irwin MR, Perlis ML. Insomnia symptoms predict longer COVID-19 symptom duration. Sleep Med. 2023;101:365–72. 10.1016/j.sleep.2022.11.019. 36493657 10.1016/j.sleep.2022.11.019 PMC9682867

[64] Irwin MR, Olmstead R, Breen EC, Witarama T, Carrillo C, Sadeghi N, et al. Cognitive behavioral therapy and Tai Chi reverse cellular and genomic markers of inflammation in late-life insomnia: a randomized controlled trial. Biol Psychiatry. 2015;78:721–9. 10.1016/j.biopsych.2015.01.010. 25748580 10.1016/j.biopsych.2015.01.010 PMC4524803

[65] Irwin MR, Hoang D, Olmstead R, Sadeghi N, Breen EC, Bower JE, et al. Tai Chi compared with cognitive behavioral therapy and the reversal of systemic, cellular and genomic markers of inflammation in breast cancer survivors with insomnia: a randomized clinical trial. Brain Behav Immun. 2024;120:159–66. 10.1016/j.bbi.2024.05.022. 38777285 10.1016/j.bbi.2024.05.022

[66] Beurel E, Toups M, Nemeroff CB. The bidirectional relationship of depression and inflammation: double trouble. Neuron. 2020;107:234–56. 10.1016/J.NEURON.2020.06.002. 32553197 10.1016/j.neuron.2020.06.002 PMC7381373

[67] Köhler CA, Freitas TH, Maes M, de Andrade NQ, Liu CS, Fernandes BS, et al. Peripheral cytokine and chemokine alterations in depression: a meta-analysis of 82 studies. Acta Psychiatr Scand. 2017;135:373–87. 10.1111/ACPS.12698. 28122130 10.1111/acps.12698

[68] Dooley LN, Kuhlman KR, Robles TF, Eisenberger NI, Craske MG, Bower JE. The role of inflammation in core features of depression: insights from paradigms using exogenously-induced inflammation. Neurosci Biobehav Rev. 2018;94:219–37. 10.1016/J.NEUBIOREV.2018.09.006. 30201219 10.1016/j.neubiorev.2018.09.006 PMC6192535

[69] Kuhlman KR, Robles TF, Dooley LN, Boyle CC, Haydon MD, Bower JE. Within-subject associations between inflammation and features of depression: using the flu vaccine as a mild inflammatory stimulus. Brain Behav Immun. 2018;69:540–7. 10.1016/J.BBI.2018.02.001. 29458196 10.1016/j.bbi.2018.02.001 PMC5857469

[70] Brydon L, Harrison NA, Walker C, Steptoe A, Critchley HD. Peripheral inflammation is associated with altered Substantia Nigra activity and psychomotor slowing in humans. Biol Psychiatry. 2008;63:1022–9. 10.1016/j.biopsych.2007.12.007. 18242584 10.1016/j.biopsych.2007.12.007 PMC2885493

[71] Reichenberg A, Yirmiya R, Schuld A, Kraus T, Haack M, Morag A, et al. Cytokine-associated emotional and cognitive disturbances in humans. Arch Gen Psychiatry. 2001;58:445–52. 10.1001/archpsyc.58.5.445. 11343523 10.1001/archpsyc.58.5.445

[72] Manigault AW, Ganz PA, Irwin MR, Cole SW, Kuhlman KR, Bower JE. Moderators of inflammation-related depression: a prospective study of breast cancer survivors. Transl Psychiatry. 2021;11(1):1. 10.1038/s41398-021-01744-6. 34873150 10.1038/s41398-021-01744-6 PMC8648787

[73] Irwin MR. Insomnia and inflammation conspire to heighten depression risk: implications for treatment and prevention of mood disorders. Biol Psychiatry. 2025;98:819–29. 10.1016/j.biopsych.2025.04.018. 40328368 10.1016/j.biopsych.2025.04.018 PMC13528946

[74] Slavich GM, Irwin MR. From stress to inflammation and major depressive disorder: a social signal transduction theory of depression. Psychol Bull. 2014;140:774–815. 10.1037/a0035302. 24417575 10.1037/a0035302 PMC4006295

[75] Dimidjian S, Goodman SH, Sherwood NE, et al. A pragmatic randomized clinical trial of behavioral activation for depressed pregnant women. J Consult Clin Psychol. 2017;85(1):26–36. 10.1037/ccp0000151. 28045285 10.1037/ccp0000151 PMC5699449

[76] Cuijpers P, Karyotaki E, Harrer M, Stikkelbroek Y. Individual behavioral activation in the treatment of depression: a meta analysis. Psychother Res. 2023;33(7):886–97. 10.1080/10503307.2023.2197630. 37068380 10.1080/10503307.2023.2197630

[77] Adamson BC, Yang Y, Motl RW. Association between compliance with physical activity guidelines, sedentary behavior and depressive symptoms. Prev Med. 2016;91:152–7. 10.1016/j.ypmed.2016.08.020. 27527574 10.1016/j.ypmed.2016.08.020

[78] Santini ZI, Koyanagi A, Tyrovolas S, Mason C, Haro JM. The association between social relationships and depression: a systematic review. J Affect Disord. 2015;175:53–65. 10.1016/j.jad.2014.12.049. 25594512 10.1016/j.jad.2014.12.049

[79] Garfield V, Llewellyn CH, Kumari M. The relationship between physical activity, sleep duration and depressive symptoms in older adults: The English Longitudinal Study of Ageing (ELSA). Prev Med Rep. 2016;4:512–6. 10.1016/j.pmedr.2016.09.006. 27699145 10.1016/j.pmedr.2016.09.006 PMC5045946

[80] Kim SY, Jeon SW, Shin DW, Oh KS, Shin YC, Lim SW. Association between physical activity and depressive symptoms in general adult populations: an analysis of the dose-response relationship. Psychiatry Res. 2018;269:258–63. 10.1016/j.psychres.2018.08.076. 30170283 10.1016/j.psychres.2018.08.076

[81] Chu C, Buchman-Schmitt JM, Stanley IH, Hom MA, Tucker RP, Hagan CR, et al. The interpersonal theory of suicide: A systematic review and meta-analysis of a decade of cross-national research. Psychol Bull US: Am Psychol Association. 2017;143:1313–45. 10.1037/bul0000123. 10.1037/bul0000123 PMC5730496 29072480

[82] Hom MA, Chu C, Rogers ML, Joiner TE. A meta-analysis of the relationship between sleep problems and loneliness. Clin Psychol Sci. 2020;8:799–824. 10.1177/2167702620922969.

[83] Werneck AO, Collings PJ, Barboza LL, Stubbs B, Silva DR. Associations of sedentary behaviors and physical activity with social isolation in 100,839 school students: The Brazilian Scholar Health Survey. Gen Hosp Psychiatry. 2019;59:7–13. 10.1016/j.genhosppsych.2019.04.010. 31054464 10.1016/j.genhosppsych.2019.04.010

[84] Wernhart S, Weihe E, Rassaf T. Reduced physical activity and weight gain are associated with an increase of depressive symptoms during the COVID-19 pandemic. A general practitioners’ prospective observational study. JRSM Cardiovasc Dis. 2021;10:20480040211047742. 10.1177/20480040211047742. 34631041 10.1177/20480040211047742 PMC8495516

[85] Wipfli B, Landers D, Nagoshi C, Ringenbach S. An examination of serotonin and psychological variables in the relationship between exercise and mental health. Scand J Med Sci Sports. 2011;21:474–81. 10.1111/j.1600-0838.2009.01049.x. 20030777 10.1111/j.1600-0838.2009.01049.x

[86] Harmer CJ. Serotonin and emotional processing: does it help explain antidepressant drug action? Neuropharmacology. 2008;55:1023–8. 10.1016/j.neuropharm.2008.06.036. 18634807 10.1016/j.neuropharm.2008.06.036

[87] Droste SK, Gesing A, Ulbricht S, Müller MB, Linthorst ACE, Reul JMHM. Effects of long-term voluntary exercise on the mouse hypothalamic-pituitary-adrenocortical axis. Endocrinology. 2003;144:3012–23. 10.1210/en.2003-0097. 12810557 10.1210/en.2003-0097

[88] Benz F, Knoop T, Ballesio A, et al. The efficacy of cognitive and behavior therapies for insomnia on daytime symptoms: a systematic review and network meta-analysis. Clin Psychol Rev. 2020;80:101873. 10.1016/j.cpr.2020.101873. 32777632 10.1016/j.cpr.2020.101873

[89] Sochal M, Feige B, Spiegelhalder K, Ell J. The effects of cognitive behavioral therapy for insomnia on physical activity before and after time in bed among shift workers. J Clin Med. 2025;14(9):3206. 10.3390/jcm14093206. 40364237 10.3390/jcm14093206 PMC12072739

[90] Tang NKY, Saconi B, Jansson-Fröjmark M, Ong JC, Carney CE. Cognitive factors and processes in models of insomnia: a systematic review. J Sleep Res. 2023;32(6):e13923. 10.1111/jsr.13923. 37364869 10.1111/jsr.13923 PMC10909484

[91] Ballesio A, Bacaro V, Vacca M, et al. Does cognitive behaviour therapy for insomnia reduce repetitive negative thinking and sleep-related worry beliefs? A systematic review and meta-analysis. Sleep Med Rev. 2021;55:101378. 10.1016/j.smrv.2020.101378. 32992228 10.1016/j.smrv.2020.101378

[92] Harvey AG. A cognitive model of insomnia. Behav Res Ther. 2002;40:869–93. 10.1016/S0005-7967(01)00061-4. 12186352 10.1016/s0005-7967(01)00061-4

[93] Ballesio A, Bacaro V, Vacca M, Chirico A, Lucidi F, Riemann D, et al. Does cognitive behaviour therapy for insomnia reduce repetitive negative thinking and sleep-related worry beliefs? A systematic review and meta-analysis. Sleep Med Rev. 2021;55:101378. 10.1016/j.smrv.2020.101378. 32992228 10.1016/j.smrv.2020.101378

[94] Norell-Clarke A, Tillfors M, Jansson-Fröjmark M, Holländare F, Engström I. How does cognitive behavioral therapy for insomnia work? An investigation of cognitive processes and time in bed as outcomes and mediators in a sample with insomnia and depressive symptomatology. Int J Cogn Ther. 2017;10:304–29. 10.1521/ijct.2017.10.4.304.

[95] Cheng P, Kalmbach DA, Castelan AC, Murugan N, Drake CL. Depression prevention in digital cognitive behavioral therapy for insomnia: is rumination a mediator? J Affect Disord. 2020;273:434–41. 10.1016/j.jad.2020.03.184. 32560938 10.1016/j.jad.2020.03.184

[96] Lau PH, Carney AE, Marway OS, Carmona NE, Amestoy M, Carney CE. Investigating the antidepressant effects of CBT-I in those with major depressive and insomnia disorders. J Affect Disord Rep. 2022;9:100366. 10.1016/j.jadr.2022.100366.

[97] Li SH, Corkish B, Richardson C, Christensen H, Werner-Seidler A. The role of rumination in the relationship between symptoms of insomnia and depression in adolescents. J Sleep Res. 2024;33:e13932. 10.1111/jsr.13932. 37198139 10.1111/jsr.13932

[98] Kyle SD, Hurry MED, Emsley R, Marsden A, Omlin X, Juss A, et al. The effects of digital cognitive behavioral therapy for insomnia on cognitive function: a randomized controlled trial. Sleep. 2020;43:zsaa034. 10.1093/sleep/zsaa034. 32128593 10.1093/sleep/zsaa034

[99] Miró E, Lupiáñez J, Martínez MP, Sánchez AI, Díaz-Piedra C, Guzmán MA, et al. Cognitive-behavioral therapy for insomnia improves attentional function in fibromyalgia syndrome: a pilot, randomized controlled trial. J Health Psychol. 2011;16:770–82. 10.1177/1359105310390544. 21346020 10.1177/1359105310390544

[100] Wagner S, Helmreich I, Wollschläger D, Meyer K, Kaaden S, Reiff J, et al. Early improvement of executive test performance during antidepressant treatment predicts treatment outcome in patients with Major Depressive Disorder. PLoS One. 2018;13:e0194574. 10.1371/journal.pone.0194574. 29668746 10.1371/journal.pone.0194574 PMC5905973

[101] Gross JJ. The emerging field of emotion regulation: an integrative review. Rev Gen Psychol. 1998;2(3):271–99. 10.1037/1089-2680.2.3.271.

[102] Watling J, Pawlik B, Scott K, Booth S, Short MA. Sleep loss and affective functioning: more than just mood. Behav Sleep Med. 2017;15:394–409. 10.1080/15402002.2016.1141770. 27158937 10.1080/15402002.2016.1141770

[103] Gujar N, Yoo S-S, Hu P, Walker MP. Sleep deprivation amplifies reactivity of brain reward networks, biasing the appraisal of positive emotional experiences. J Neurosci. 2011;31:4466–74. 10.1523/JNEUROSCI.3220-10.2011. 21430147 10.1523/JNEUROSCI.3220-10.2011 PMC3086142

[104] Yoo S-S, Gujar N, Hu P, Jolesz FA, Walker MP. The human emotional brain without sleep–a prefrontal amygdala disconnect. Curr Biol. 2007;17:R877–878. 10.1016/j.cub.2007.08.007. 17956744 10.1016/j.cub.2007.08.007

[105] Tempesta D, Couyoumdjian A, Curcio G, Moroni F, Marzano C, De Gennaro L, et al. Lack of sleep affects the evaluation of emotional stimuli. Brain Res Bull. 2010;82:104–8. 10.1016/j.brainresbull.2010.01.014. 20117179 10.1016/j.brainresbull.2010.01.014

[106] Baglioni C, Spiegelhalder K, Lombardo C, Riemann D. Sleep and emotions: a focus on insomnia. Sleep Medicine Reviews. 2010;14(4):227–38. 10.1016/j.smrv.2009.10.007. 20137989 10.1016/j.smrv.2009.10.007

[107] Klumpp H, Roberts J, Kapella MC, Kennedy AE, Kumar A, Phan KL. Subjective and objective sleep quality modulate emotion regulatory brain function in anxiety and depression. Depression and Anxiety. 2017;34(7):651–60. 10.1002/da.22622. 28419607 10.1002/da.22622 PMC5503154

[108] O’Leary K, Bylsma LM, Rottenberg J. Why might poor sleep quality lead to depression? A role for emotion regulation. Cognition & Emotion. 2017;31(8):1698–706. 10.1080/02699931.2016.1247035. 27807996 10.1080/02699931.2016.1247035 PMC6190702

[109] Tamm S, Tse KYK, Hellier J, Saunders KEA, Harmer CJ, Espie CA, et al. Emotional processing following digital cognitive behavioral therapy for insomnia in people with depressive symptoms: a randomized clinical trial. JAMA Netw Open. 2025;8:e2461502. 10.1001/jamanetworkopen.2024.61502. 40014347 10.1001/jamanetworkopen.2024.61502 PMC11868973

[110] Naragon-Gainey K, McMahon TP, Chacko TP. The structure of common emotion regulation strategies: a meta-analytic examination. Psychol Bull. 2017;143:384–427. 10.1037/bul0000093. 28301202 10.1037/bul0000093

[111] Zakiei A, Khazaie H, Reshadat S, Rezaei M, Komasi S. The comparison of emotional dysregulation and experiential avoidance in patients with insomnia and non-clinical population. J Caring Sci. 2020;9:87–92. 10.34172/JCS.2020.013. 32626670 10.34172/JCS.2020.013 PMC7322408

[112] Scotta AV, Cortez MV, Miranda AR. Insomnia is associated with worry, cognitive avoidance and low academic engagement in Argentinian university students during the COVID-19 social isolation. Psychol Health Med. 2022;27:199–214. 10.1080/13548506.2020.1869796. 33382639 10.1080/13548506.2020.1869796

[113] Cheng M-Y, Wang M-J, Chang M-Y, Zhang R-X, Gu C-F, Zhao Y-H. Relationship between resilience and insomnia among the middle-aged and elderly: mediating role of maladaptive emotion regulation strategies. Psychol Health Med. 2020;25:1266–77. 10.1080/13548506.2020.1734637. 32098490 10.1080/13548506.2020.1734637

[114] Tomaso CC, Johnson AB, Nelson TD. The effect of sleep deprivation and restriction on mood, emotion, and emotion regulation: three meta-analyses in one. Sleep. 2021;44:zsaa289. 10.1093/sleep/zsaa289. 33367799 10.1093/sleep/zsaa289 PMC8193556

[115] Byrne L, Donovan C, Shiels A. Recharge: a preliminary evaluation of an emotion regulation enhanced CBT-i intervention for insomnia in early adolescence. Behav Cogn Psychother. 2020;48:121–6. 10.1017/S1352465819000481. 31379309 10.1017/S1352465819000481

[116] Moukhtarian TR, Fletcher S, Walasek L, Patel K, Toro C, Hurley-Wallace AL, et al. Digital CBT for insomnia and emotion regulation in the workplace: a randomised waitlist-controlled trial. Psychol Med. 2025;55:e52. 10.1017/S0033291725000194. 39957531 10.1017/S0033291725000194 PMC12080640

[117] Gujar N, Yoo S-S, Hu P, Walker MP. Sleep deprivation amplifies reactivity of brain reward networks, biasing the appraisal of positive emotional experiences. J Neurosci. 2011;31(12):4466–74. 10.1523/JNEUROSCI.3220-10.2011. 21430147 10.1523/JNEUROSCI.3220-10.2011 PMC3086142

[118] Tempesta D, Couyoumdjian A, Curcio G, et al. Lack of sleep affects the evaluation of emotional stimuli. Brain Res Bull. 2010;82(1–2):104–8. 10.1016/j.brainresbull.2010.01.014. 20117179 10.1016/j.brainresbull.2010.01.014

[119] Yoo S-S, Gujar N, Hu P, Jolesz FA, Walker MP. The human emotional brain without sleep–a prefrontal amygdala disconnect. Curr Biology: CB. 2007;17:20. 10.1016/j.cub.2007.08.007. 10.1016/j.cub.2007.08.007 17956744

[120] Tomaso CC, Johnson AB, Nelson TD. The effect of sleep deprivation and restriction on mood, emotion, and emotion regulation: three meta-analyses in one. Sleep. 2021;44(6):zsaa289. 10.1093/sleep/zsaa289. 33367799 10.1093/sleep/zsaa289 PMC8193556

[121] Ballesio A, Bacaro V, Vacca M, et al. Does cognitive behaviour therapy for insomnia reduce repetitive negative thinking and sleep-related worry beliefs? A systematic review and meta-analysis. Sleep Med Rev. 2021;55:101378. 10.1016/j.smrv.2020.101378. 32992228 10.1016/j.smrv.2020.101378

[122] Li SH, Corkish B, Richardson C, Christensen H, Werner-Seidler A. The role of rumination in the relationship between symptoms of insomnia and depression in adolescents. J Sleep Res. 2024;33(2):e13932. 10.1111/jsr.13932. 37198139 10.1111/jsr.13932

